# Analysis of chemiluminescence and liquid chromatography-mass spectrometry in 25-hydroxyvitamin D detection using fuzzy logic

**DOI:** 10.1038/s41598-026-41793-9

**Published:** 2026-03-03

**Authors:** Hongkun Liu, Sirui Li, Kok Wai Wong, Yujie Li, Xinyi He, Shuhao Liang, Hui Deng, Linlin Zhang, Lei Zhang, Jianli Cui

**Affiliations:** 1 Department of Clinical laboratory, Sichuan Taikang Hospital, Chengdu, Sichuan P. R. China; 2 School of Information Technology, Murdoch University, 90 South Street, Perth, WA 6150 Australia; 3 College of Life Science, Longyan University, Longyan, Fujian P. R. China

**Keywords:** Fuzzy Inference System, 25-Hydroxyvitamin D (25(OH)D), Chemiluminescence immunoassay (CLIA), Liquid chromatography-tandem mass spectrometry (LC-MS/MS), Biochemistry, Biomarkers, Diseases, Endocrinology, Health care, Medical research

## Abstract

Vitamin D is an essential nutrient closely associated with the prevention of multiple diseases, including osteoporosis, diabetes, and cardiovascular disorders. The serum level of 25-hydroxyvitamin D [25(OH)D] is the primary biomarker for assessing vitamin D status, and its precise quantification is critical for clinical diagnosis and treatment. Chemiluminescence immunoassay (CLIA) and liquid chromatography–tandem mass spectrometry (LC-MS/MS) are widely used analytical methods; however, methodological discrepancies often lead to inconsistent results that may be further influenced by demographic factors such as age and gender. In this study, we evaluated the consistency and correlation between CLIA and LC-MS/MS measurements using a Generative Fuzzy Inference System (GENFIS). Analysis of 138 serum samples showed that LC-MS/MS produced significantly higher 25(OH)D concentrations than CLIA (*p* < 0.01; mean difference = 1.33 ± 3.71; 95% CI: − 5.95 to 8.61), though the two methods exhibited strong linear correlation and agreement (Cohen’s Kappa = 0.8257; R² = 0.9075; intraclass correlation coefficient = 0.93). GENFIS analysis indicated a possible 30–40-year female pattern of larger between the detection differences of two methods. To verify this finding, an additional 59 samples were analyzed, revealing a relative risk (RR) of 3.18 (95% CI: 1.71–5.93; *p* < 0.05) for this subgroup compared with other populations, supporting the GENFIS inference. These results highlight age- and gender-related differences in 25(OH)D measurement between CLIA and LC-MS/MS, providing valuable insights for improving the standardization and clinical interpretation of vitamin D testing.

**Supplementary Information:**

The online version contains supplementary material available at 10.1038/s41598-026-41793-9.

## Introduction

 Vitamins are essential nutrients vital to physiological function and overall health. Among them, vitamin D has attracted considerable attention for its role in maintaining bone integrity, modulating immune responses, and preventing a range of diseases^1^. Recent studies^2,3^ suggest that adequate vitamin D levels may reduce the risk of conditions such as Alzheimer’s disease, respiratory infections in infants, and asthma in offspring when supplemented during pregnancy. Additionally, insufficient vitamin D may result in overactivation of the adaptive immune system, thereby increasing the risk of diseases such as type 1 diabetes and atherosclerosis^1,4^. Despite its importance, vitamin D deficiency remains widespread globally, particularly in developing regions and among low-income populations^5,6^, imposing a considerable burden on public health systems. Therefore, accurate and timely assessment of vitamin D status is essential to guide supplementation and support preventive healthcare. Testing of serum 25(OH)D has been the focus of global harmonization efforts through the Vitamin D Standardization Program (VDSP), which promotes traceability to the reference measurement procedures listed by the International Federation of Clinical Chemistry and Laboratory Medicine (IFCC) and the Joint Committee for Traceability in Laboratory Medicine (JCTLM), and calibration against National Institute of Standards and Technology (NIST) standard reference materials^7^.

Serum 25-hydroxyvitamin D [25(OH)D] concentration is recognized as the most reliable biomarker of vitamin D status^1^. Among the available analytical methods, chemiluminescence immunoassay (CLIA) is widely used in clinical laboratories and population screenings due to its simplicity, high throughput, automation, and efficiency^8,9^. Electrochemiluminescence immunoassay (ECLIA), an advanced form of CLIA, further enhances sensitivity, reproducibility, and detection range^10^. However, CLIA results may vary across laboratories and regions because of inconsistent calibration systems and standardization^8^. Moreover, CLIA is prone to antibody cross-reactivity and cannot differentiate between vitamin D₂and D₃^8,9^.

Recently, Liquid chromatography–tandem mass spectrometry (LC-MS/MS) has been recognised as the gold standard for 25(OH)D testing^11^, addresses some of CLIA’s limitations—particularly its ability to distinguish between vitamin D₂ and D₃. Yet, LC-MS/MS also presents challenges: it demands expert operators^11^, and cross-reactivity from vitamin D metabolites can interfere with total 25(OH)D measurement^8^. For instance, Fraser and Milan^12^ reported that vitamin D₂ can affect the accuracy of results. Given the complementary strengths and limitations of these methods, a systematic comparison is necessary to improve measurement reliability and standardisation.

Although previous studies have compared CLIA and LC-MS/MS, most have focused on specific subpopulations—such as children—and their findings have been inconsistent or even contradictory^8,13,14^. These discrepancies suggest that the factors underlying inter-method differences are not fully understood. Expanding the study population and integrating advanced analytical approaches, such as artificial intelligence (AI)-based models, may help elucidate these complex relationships and improve standardization efforts.

**Fig. 1:**
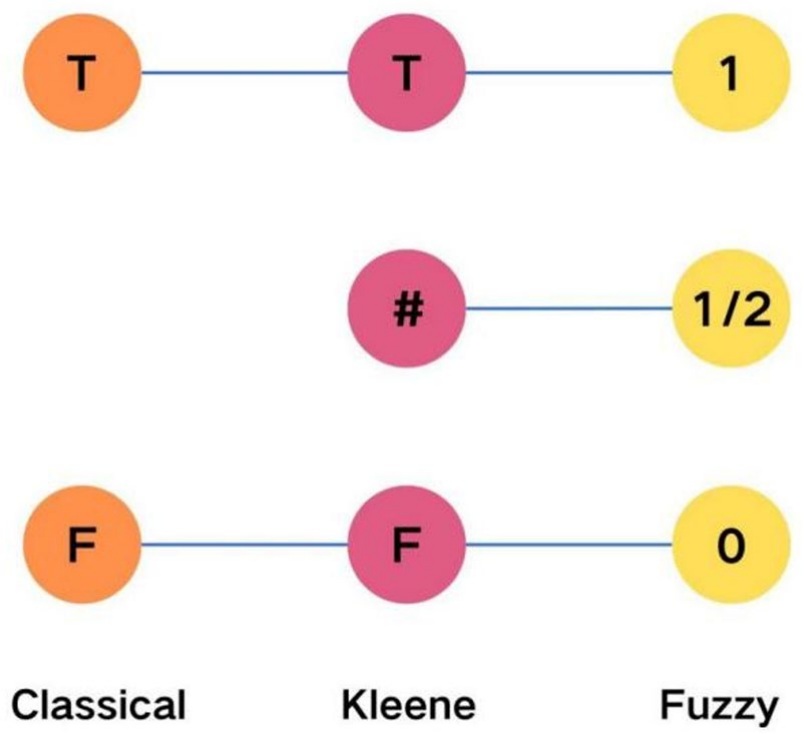
The differences between classical logic and fuzzy logic; *In classical logic*,* decision boundaries are typically clear and well-defined*,* as shown in the left part of the figure (classical)*,* where the boundary distinctly separates true (T*,* 1) and false (F*,* 0). In fuzzy logic*,* decision boundaries are usually ambiguous*,* as illustrated in the right*.

With rapid advances in AI, its applications in biomedical research are growing rapidly. Although AI has been scarcely applied to vitamin D analysis, it holds great promise for improving interpretability and managing uncertainty. Differences between CLIA and LC-MS/MS measurements may be affected by nonlinear factors such as individual variability and matrix effects—sources of uncertainty that traditional statistical methods struggle to address. AI techniques, particularly those based on fuzzy logic, are well-suited to handle such vagueness and nonlinear relationships^15^. While neural network models dominate current AI research, they typically require large datasets and often lack interpretability under uncertain conditions^15^. In contrast, fuzzy logic is effective with smaller datasets and can integrate expert knowledge, making it particularly suitable for studies involving ambiguous variables and limited data^15,16^. Unlike classical logic, which operates on binary true–false decisions, fuzzy logic accommodates degrees of truth (Fig. 1), allowing nuanced modeling of real-world biological phenomena^16^.

In medical and life sciences, many analytical concepts are inherently uncertain. For example, in differential analysis, statements such as “matrix effects may affect measurement accuracy” contain vague qualifiers that traditional models cannot quantify. Liu et al.^17^ demonstrated that fuzzy logic–based AI models can effectively mitigate such uncertainties. Fuzzy logic has been successfully applied to various domains of biomedical data analysis and differential diagnosis^18,19^. For instance, Taylan et al.^19^ used fuzzy neural inference to assess clinical differences among 17 cardiovascular diseases, illustrating its strong potential in medical data interpretation^20^.

In summary, to improve differential analysis between CLIA and LC-MS/MS, this study measures serum 25(OH)D levels in a diverse population under defined inclusion and exclusion criteria using advanced detection instruments. By integrating fuzzy inference modeling with conventional data analysis, this research aims to uncover interpretable patterns underlying method discrepancies, providing new insights for standardizing vitamin D detection and enhancing clinical decision-making.

## Materials and methods

### Datasets

This study utilized the datasets obtained from Sichuan Taikang Hospital, with formal permission granted by the hospital’s authorized personnel. The data were retrospectively and randomly selected from a diverse population who underwent health checkups and received 25(OH)D testing by chemiluminescence immunoassay (CLIA) between October 2024 and February 2025. On the same day, these samples were reanalyzed using liquid chromatography–tandem mass spectrometry (LC-MS/MS) at the Precision Laboratory of the hospital’s Medical Laboratory Department.

The dataset excluded inpatients and individuals with cardiovascular diseases, severe immune deficiencies, or major infectious diseases. All personal identifiers were removed before analysis, ensuring complete anonymity and eliminating privacy concerns.

CLIA testing was performed using the Roche cobas e 801 analyzer with the Elecsys Vitamin D total II kit (Roche Diagnostics GmbH). The assay is based on a competitive binding principle, with a quantification range of 3–100 ng/mL. LC-MS/MS analyses were conducted using the AB Sciex QTRAP 6500 instrument, with reagents provided by Guangdong Zhongke Qingzi Medical Technology Co., Ltd. All procedures followed manufacturer protocols, and quality control was performed for each analytical batch. The detection instruments used in this study are certified by the China Food and Drug Administration (CFDA) and represent the most advanced testing systems currently approved in China.

### Data analysis

Data were processed and analyzed using MATLAB R2020b, Microsoft Excel 2016, and PyCharm 2022.3. Linear regression analysis was used to evaluate the correlation between CLIA and LC-MS/MS measurements, while a paired t-test assessed the mean difference between the two methods for the same cohort. The regression equation is expressed as:

1 $$f\left( x \right) = p1x + p2$$

where f(x) indicates the dependent variable (LC-MS/MS testing results), x represents the independent variable (CLIA testing results), p1 is the slope, and p2 is the intercept.

The agreement between CLIA and LC-MS/MS is evaluated employing two complementary methods. First, the intraclass correlation coefficient (ICC) is calculated to assess measurement consistency, where ICC < 0.40 indicates poor consistency and ICC > 0.75 means excellent consistency.The ICC is calculated from between-group (MSB) and within-group (MSW) variances according to the formula:

2 $$ICC{\text{ }} = {\text{ }}\left( {MSB{\text{ }} - {\text{ }}MSW} \right){\text{ }}/{\text{ }}\left( {MSB{\text{ }} + {\text{ }}\left( {k - 1} \right){\text{ }} \times {\text{ }}MSW} \right)$$

where k represents the number of evaluated columns. Since this study assessed the result differences between two testing methods, k is equal to 2.

Second, the Bland-Altman analysis was performed to assess the mean bias and the limits of agreement (LOA) between the two testing methods. The LOA were calculated as:

3 $$LOA{\text{ }} = {\text{ }}Mean{\text{ }}difference \pm 1.96 \times SD$$

where SD represents the standard deviation of the differences. This method allows illustration of systematic bias between CLIA and LC-MS/MS results.

The classification consistency between the two methods was evaluated utilizing Cohen’s Kappa analysis, with a Kappa coefficient > 0.8 indicating strong consistency. The collected quantitative data were expressed as mean ± standard deviation (x̄±s).

### Fuzzy logic analysis

In biomedical analyses, relationships among variables are often nonlinear and influenced by uncertain factors such as age and gender. Fuzzy logic offers a powerful framework to address such uncertainties by allowing continuous rather than binary reasoning. Its flexibility enables adjustment of variable types and weights, thereby enhancing the interpretability of complex patterns underlying measurement differences.

This study employed a Generative Fuzzy Inference System (GENFIS) integrated with Fuzzy C-Means (FCM) clustering to automatically generate fuzzy rules from the data. The FCM algorithm partitions the dataset into n clusters, assigning each data point a membership degree for each cluster. The objective function is given by:

4 $$\:{J}_{m}={\sum\:}_{i=1}^{D}\:{\sum\:}_{j=1}^{N}\:{\mu\:}_{ij}^{m}\Vert\:xi-cj\Vert\:^2$$

where D denotes the total number of data points, N the number of clusters, m > 1 the fuzzy overlap parameter controlling the degree of cluster fuzziness, x_i_ the ith data point, cⱼ the center of the jth cluster, and µ_i_ⱼ the membership degree of x_i_ in cluster j^21^. Following standard practice for biomedical modeling, the number of clusters (n) was set to 3 and the overlap parameter (m) to 2.

**Fig. 2:**
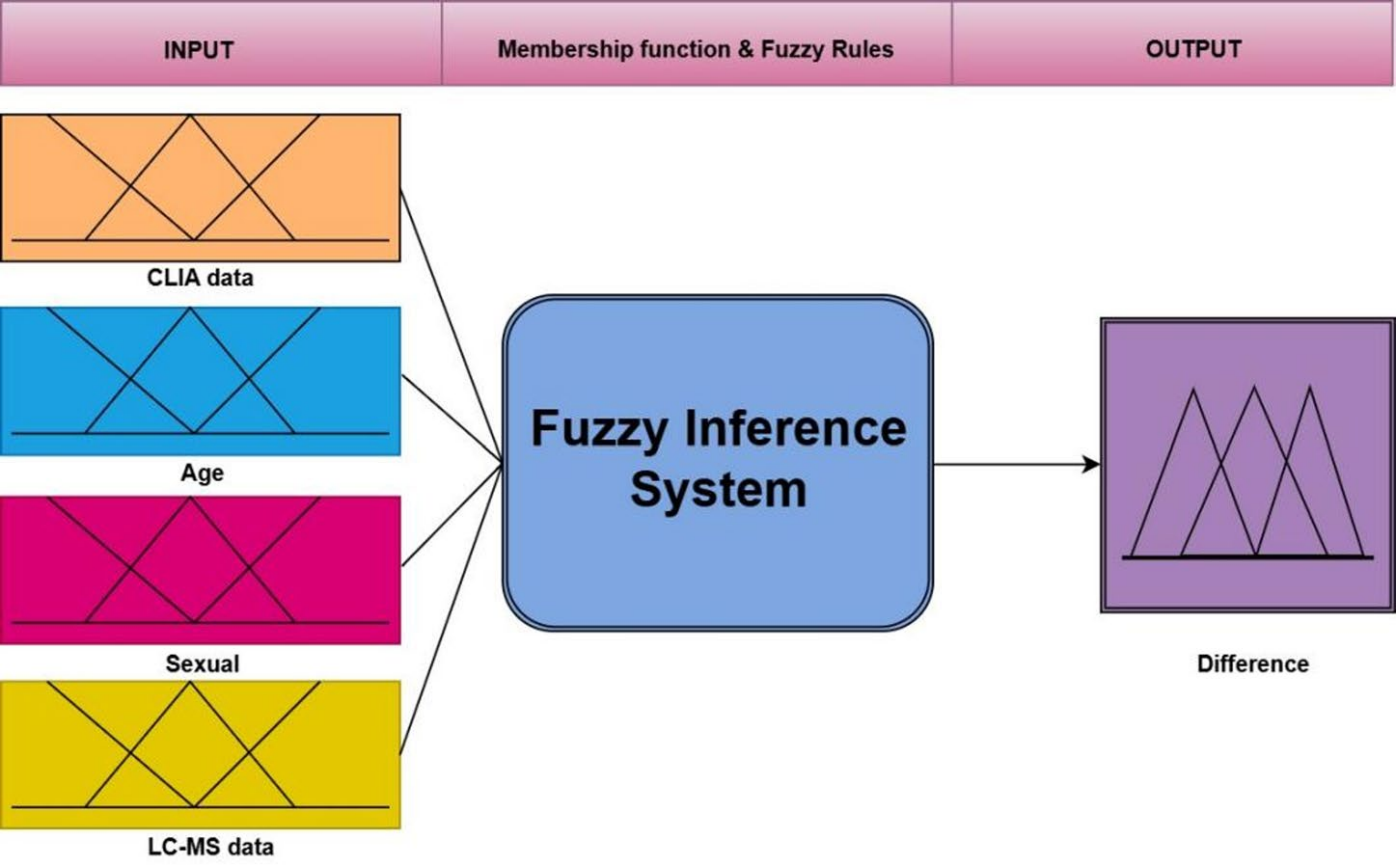
The structure of fuzzy logic model.

In this model, CLIA and LC-MS/MS results, age, and gender were input variables, while the difference between CLIA and LC-MS/MS measurements was defined as the output variable. The structure of the fuzzy inference system is illustrated in Fig. 2. All fuzzy modeling was conducted using MATLAB R2020b.

GENFIS summarizes the relationships between inputs (CLIA, LC-MS/MS, age, sex) and the output (method difference) through interpretable IF–THEN rules. FCM identifies graded data patterns, which GENFIS then translates into weighted fuzzy rules that quantify how specific input combinations contribute to observed inter-method variations. This approach enhances explainability while maintaining predictive precision.

## Results

### Samples collection & testing results

**Fig. 3:**
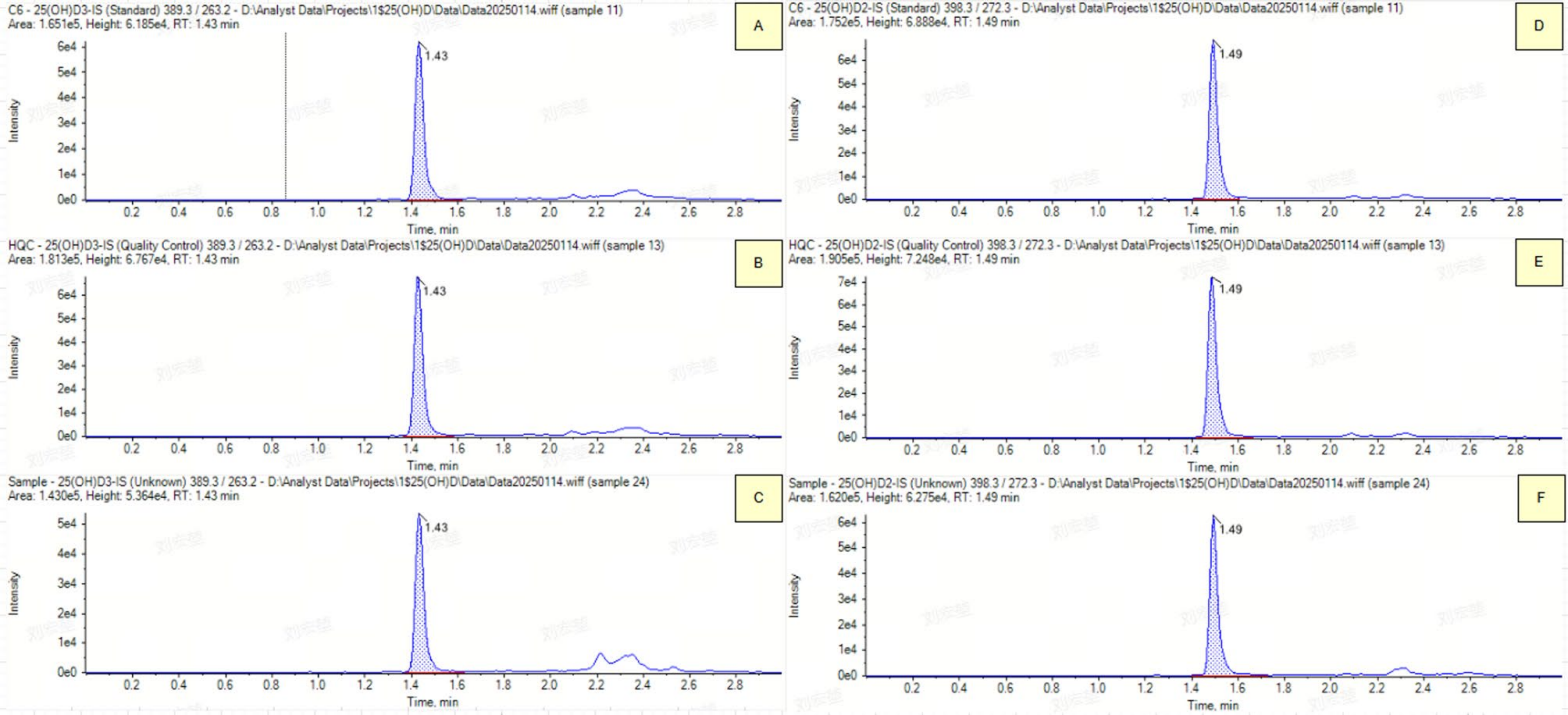
LC-MS/MS Chromatograms of Internal Standards in Different Samples; *Subfigures A*,* B*,* and C display the chromatograms of the internal standard 25(OH)D3*,* while subfigures D*,* E*,* and F display the chromatograms of the internal standard 25(OH)D2.*

According to the inclusion and exclusion criteria outlined above (Sect. 2.1), this study collected a total of 138 samples, including 60 males (43.5%) and 78 females (56.5%), with a mean age of 40.75 ± 15.59 (Table 1). The detection range of CLIA is from 3.0 ng/mL to 55.2 ng/mL, with a mean of 20.71 ± 9.22 ng/mL; the testing range of LC-MS/MS is between 5.09 ng/mL and 56.24 ng/mL, with a mean of 22.04 ± 11.23 ng/mL(Fig. 3). The difference range between the two methods is −4.56 ng/mL to 13.19 ng/mL, with a mean difference of 1.33 ± 3.71 ng/mL (Table 1).

**Table 1:** Description of Sample Information and Testing Results.

Items	Value (total number or *n* (%)/Mean + SD)
Total samples	138
Age	40.75 ± 15.59
Male	43.5% (60)
Female	56.5% (78)
CLIA (ng/mL)	20.71 ± 9.22
LC-MS/MS (ng/mL)	22.04 ± 11.23
Difference (ng/mL)	1.33 ± 3.71

### Data analysis of the testing differences

According to Table 2, the paired t-test results for the two detection methods provide a P-value < 0.05, indicating statistically significant differences. The average of the LC-MS/MS testing results are higher than those of CLIA (Table 1).

**Table 2:** The results of the bland-altman analysis and t-test.

Items	Results value
MSB	102.08
MSW	3.87
ICC	0.93
The mean difference	1.33
LOA range	−5.95-8.61
*P*-value (t-test)	< 0.01

**Fig. 4:**
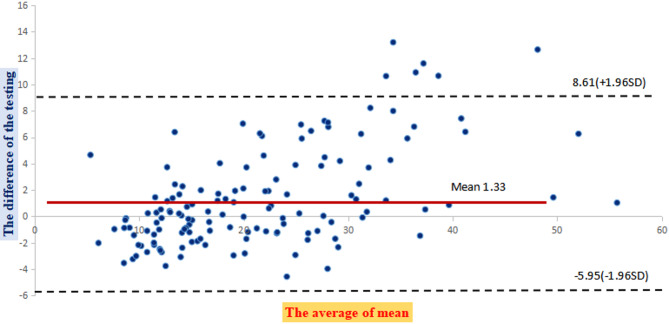
Bland-Altman analysis for the differences between the testing method of CLIA and LC-MS/MS;*The confidence interval equals the mean (1.33) plus or minus 1.96 times the standard deviation (SD). The SD of the differences is 3.71.*

The result of ICC was calculated as 0.93 (> 0.75), meaning high consistency.between the two detection methods. Bland-Altman analysis shows the mean difference of 1.33 ng/mL between LC-MS/MS and CLIA detection, with a SD of 3.71 ng/mL. The range of LOA between − 5.95 ng/mL and 8.61 ng/mL (Fig. 4).

Using the clinical threshold of 20 ng/mL for 25(OH)D deficiency as the cutoff for Cohen’s Kappa analysis^22^, the agreement rate of the two methods is calculated as the proportion of samples (126) where both methods produce results ≥ 20 ng/mL or < 20 ng/mL out of the total sample size (138), with the formula:

5 $$Agreement{\text{ }}rate{\text{ }} = {\text{ }}\left( {number{\text{ }}of{\text{ }}consistent{\text{ }}samples{\text{ }}/{\text{ }}total{\text{ }}samples} \right){\text{ }} \times {{ }}100\%$$

The calculated agreement rate is 126/138 = 91.3%, and the Cohen’s Kappa coefficient is 0.83(> 0.80), suggesting strong consistency between the two methods at the 20 ng/mL threshold (Table 3).

**Table 3:** The results of classification agreement rate between the two methods and Cohen’s Kappa.

CLIA ≥ 20 ng/mL	LC-MS/MS ≥ 20 ng/mL	LC-MS/MS < 20 ng/mL
	60 (43.48%)	5 (3.62%)
**CLIA < 20 ng/mL**	7 (5.07%)	66 (47.83%)
**Cohen’s Kappa**	0.83	

**Fig. 5:**
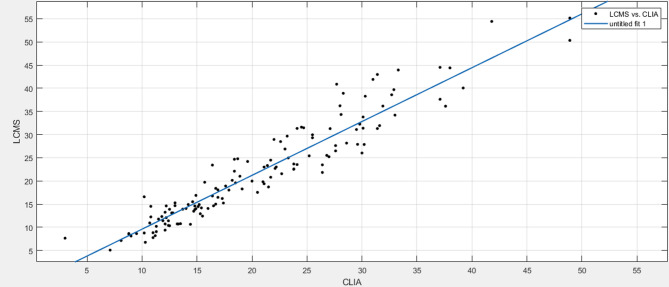
The linear relationships between the testing results of LC-MS/MS and CLIA.

Table 4; Fig. 5 show information related to the linear regression. The adjusted R² indicates a strong linear relationship, with approximately 91% of the variance in the dependent variable explained by the independent variable. The sum of squared residuals (SSE) and root mean square error (RMSE) are small, suggesting low prediction errors.

**Table 4:** The general results of linear regression analysis.

Regression Statistics	
The regression equation	LC-MS/MS = 1.161×CLIA-2.009
R²	0.9082
Adjusted R²	0.9075
Coefficients (with 95% CI)	p1 = 1.161(1.099, 1.224)
	p2=−2.009 (−3.427, −0.5901)
SSE	1586
RMSE	3.415

### Clinical threshold agreement and reclassification

Utilizing decision thresholds of 20 and 30 ng/mL^22^, cross-classification of CLIA and LC-MS/MS showed that 12/138 (8.70%; Wilson 95% CI 5.0–14.6.0.6%) individuals would change diagnostic category depending on the method. At 20 ng/mL, the number 5 and 7 (CLIA ≥ 20 and LC–MS/MS < 20 = 5; CLIA < 20 & LC-MS/MS ≥ 20 = 7). At 30 ng/mL, the number is 2 and 10 (CLIA ≥ 30 and LC–MS/MS < 30 = 2; CLIA < 30 & LC–MS/MS ≥ 30 = 12). The overall agreement was 91.3%, with Cohen’s kappa is 0.83.

### Fuzzy logic establishment and analysis

**Fig. 6:**
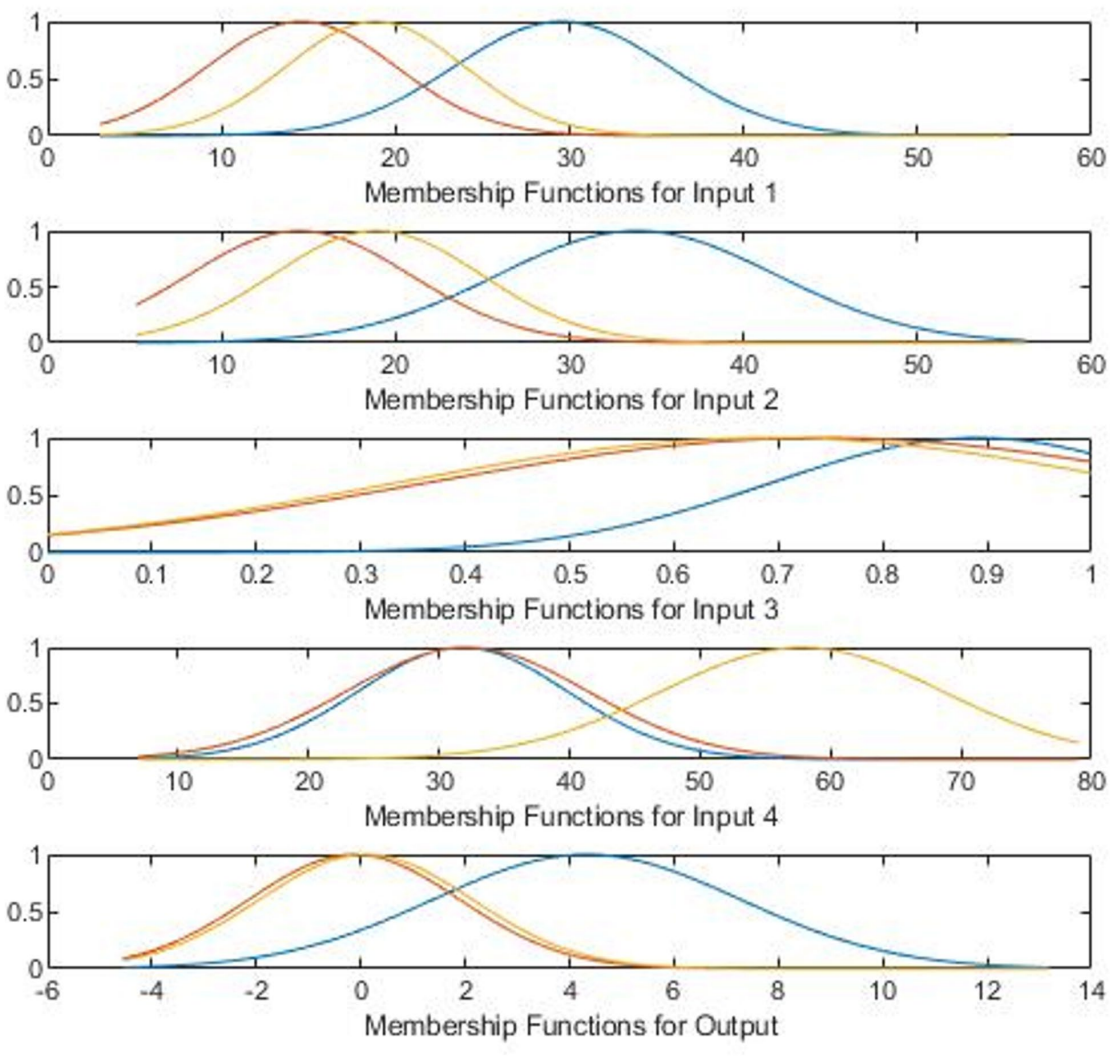
The membership function of the fuzzy logic analysis for the differences between the two testing methods. *Low is represented by the area under the blue line*,* Medium by the orange*,* and High by the yellow.*

Both the fuzzy membership functions and fuzzy rules were automatically derived from the observed input-output data rather than manually defined, ensuring an objective, data-driven rule generation process. After GENFIS identified the input and output data, it generated fuzzy membership functions and three data-driven fuzzy rules (as shown in Fig. 6), each corresponding to a cluster in the input-output space (Table 5). The input variables Input1, Input2, Input3, and Input4 represent the CLIA testing result, LC-MS/MS testing result, gender, and age, respectively, while the Output represents the differences of CLIA and LC-MS/MS testing. The differences are normalized to a range of [0, 1] based on the equation:

**Table 5:** The GENFIS rules information.

Rules	CLIA Level	LC-MS/MS Level	Gender	Age	Output (Difference)	Normalized Weight
R1	Low	Low	Female	30–40	Medium	0.386
R3	Medium	Medium	Female	40–60	Medium	0.32
R2	High	High	Female	30–40	High	0.294

6 $$output - norm = output - min/max - min$$

The following are the fuzzy rules extracted from the input-output data:

R1: If (CLIA (in1) testing results is low) and (LC-MS/MS testing results (in2) is low) and (gender (in3) is low) and (age (in4) is low) then (difference (output) is low).

R2: If (CLIA testing results is medium) and (LC-MS/MS testing results is medium) and (gender is medium) and (age is medium) then (difference is medium).

R3: If (CLIA testing result is high) and (LC-MS/MS testing result is high) and (gender is high) and (age is high) then (difference is high).

**Fig. 7:**
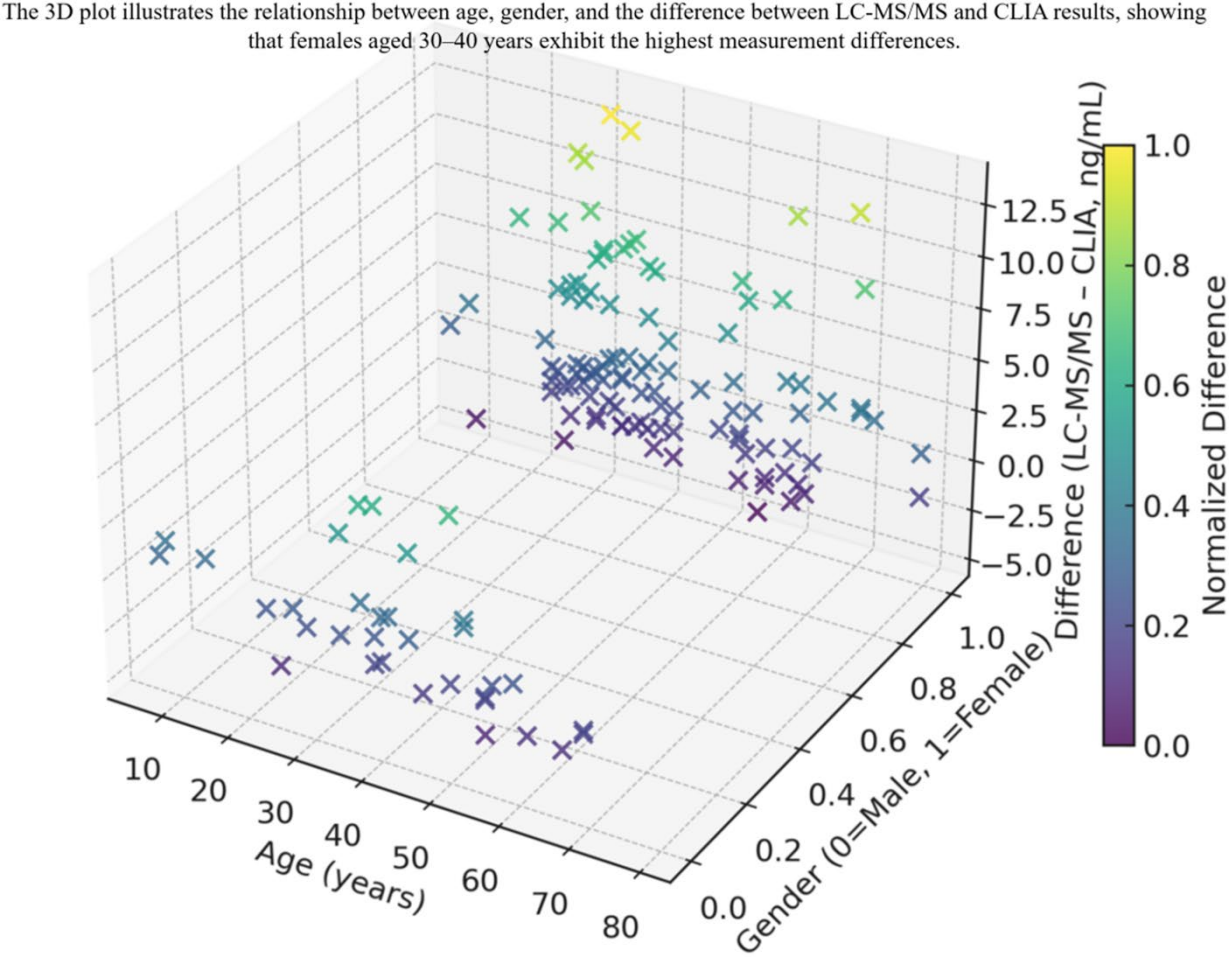
The three-dimensional plot of GEMFIS rule surface.

As a result, when 25(OH)D levels exceed the threshold (20 ng/mL), they are more likely to result in greater differences, contributing significantly to the variability. The differences across age groups in males are not significant, whereas in females, the differences are most pronounced in the 30–40 age group (Table 6). The rule surface visualization in Fig. 7 further shows that higher differences are concentrated within this demographic group.

**Table 6:** The results of fuzzy logic output in the women’s differences between different ages.

Age	The output of GENFIS
20	0.299
30	0.348
35	0.349
40	0.342
50	0.285
> 60	≦ 0.27

Note: *The detection threshold for both methods is set at 20 ng/mL; when the results of the two methods are consistent*,* the trends of the remaining results are the similar*.

### Fuzzy Reasoning Verification

To validate the output results of GENFIS, this study collected an additional 59 samples for verification. The experimental group consisted of women aged 30–40 years (28 samples), while the control group included men or women outside the 30–40 age range (31 samples). The average difference between the detection methods was calculated as 1.70 ng/ml, which was used as the threshold to classify the differences into high (≥ 1.70 ng/ml) and low (< 1.70 ng/ml) categories. Table 7 shows the proportion of high differences in the experimental group was 3.18 times that of the control group (RR = 3.18, 95% CI: 1.71–5.93). This result provides preliminary support for the GENFIS hypothesis that women aged 30–40 may amplify the differences between the detection methods. The CI excludes 1 (*p* < 0.05), indicating a statistically significant difference between the groups.

**Table 7:** Relative Risk (RR) of High Differences Between Detection Methods.

Experinental group	High difference	Low difference
	0.536 (15/28)	0.226 (7/31)
Control group	0.464 (13/28)	0.774 (24/31)
Relative Risk (RR)	3.31	
95% CI	1.71–5.93	

## Disucussion

As one of the essential fat-soluble vitamins in the human body, studies have shown that vitamin D is not only involved in bone health but also plays a key role in various diseases^23,24^. Research shows^25^ that prediabetic individuals with vitamin D levels above the sufficiency threshold (25(OH)D > 30ng/ml) have a 38% lower risk of developing type 2 diabetes (T2D) compared to those with severe deficiency (25(OH)D < 12ng/ml). During the COVID-19 pandemic, vitamin D has played a positive role in preventing infections^26^. Thus, monitoring vitamin D levels is important for promoting overall health development. 25(OH)D is the primary metabolic form of vitamin D in the body, and CLIA and LC-MS/MS are the two most commonly used methods for detecting 25(OH)D^14^, each with its own advantages and limitations. Through comparing the differences between these two widely used testing methods, the consistency of 25(OH)D detection can be improved, thereby facilitating further advancements in these two detection technologies.

The results of this study indicated that the average detection values of LC-MS/MS are higher than those of CLIA, with a statistically significant difference (*P* < 0.05) (see Tables 1 and 2). This is attributed to the differing testing principles of the two methods: CLIA cannot effectively distinguish between D2 and D3, leading to overall lower detection levels compared to LC-MS/MS. The findings of this study differ from those of other related studies^8,14^, which may be due to differences in the study population, reagents, and detection equipment; this will be discussed in detail in later sections. Linear regression analysis revealed an R² > 0.90, indicating a high degree of fit between the two detection methods. Bland-Altman analysis demonstrated an ICC > 0.9 for the two methods, suggesting excellent consistency. Additionally, a Cohen’s Kappa coefficient > 0.8 indicated near-perfect agreement in the classification results of the two methods, with an agreement rate exceeding 90%.

The average 25(OH)D levels detected in this study are all below 30 ng/mL, slightly above 20 ng/mL. In some studies^25,27^, 25(OH)D levels below 30 ng/mL are considered indicative of vitamin D insufficiency. Given that medical checkups and treatments typically follow the principle of proximity, this suggests that vitamin D levels in this region still warrant attention. Other related studies^8,13,14^ have shown similar patterns in vitamin D levels, whereas the study by Biswasarma et al. revealed a more concerning situation: the average vitamin D level among women in Bangladesh was below 20 ng/mL^28^. A commonality between these studies and the present study is that they were all conducted in Asian countries, though not all of these regions are low-income areas. For example, the region in this study has a per capita Gross Domestic Product (GDP) exceeding 15,000 USD^29^, reaching the level of high-income countries. It suggests that the relatively low vitamin D levels observed in this study may be more closely associated with the region’s dietary habits and climatic conditions. The research by Lips et al.^30^ also confirmed that vitamin D levels in Asian countries are lower than those in the Americas and Europe. They proposed that this is related to the more frequent consumption of dairy products among Western populations^30^. Consequently, Asian countries should continue to monitor the vitamin D levels of their populations. Although the mean bias is small, a limited percentage of cases (8.70%, 95% CI 5.0%–14.6%) near clinical thresholds may be reclassified depending on the method used, showing some affections for threshold-based decision-making. In addition, small bias It may arise when assays are not fully aligned to IFCC reference procedures or uniformly calibrated to NIST Standard Reference Materials (SRMs)^31^. Routine participation in Vitamin D External Quality Assessment Scheme (DEQAS) and alignment with VDSP recommendations can reduce such discrepancies and mitigate threshold‑dependent reclassification. In practice, laboratories should verify traceability, monitor external quality performance, and interpret results near decision thresholds with particular caution, especially when changing methods^7^.

GENFIS algorithm produces fuzzy rules by extracting the relationships between input and output variabless—in other words, fuzzy rules are defined based on those input–output patterns rather than being manually specified—and then uses these rules to describe the relationships among fuzzy sets (clusters)^32^. Due to its ability to handle ambiguous concepts^15^, fuzzy logic is frequently applied in biomedical research. Another key feature of fuzzy logic is its capacity to perform analysis with limited data types and quantities. In light of these advantages and the project’s requirements, fuzzy logic is employed to further explore the distinctions between the two detection techniques. In this study, inference analysis was conducted based on data from 138 samples. The membership functions for age and gender reveal that all clusters tend toward 1 (female) in gender, while in age, high level overlaps significantly with low and medium in the 30–40 age range. According to GENFIS analysis, a tentative pattern of greater between the differences of two testing methods in women aged 30–40 emerged but this hypothesis-generating observation requires confirmation in larger cohorts with multivariable adjustment. In addition,, female samples outnumber male ones, and although this imbalance is modest, it may subtly influence model inference. Compared with traditional methods^8^, which typically rely on linear relationships, fuzzy logic is more capable of capturing ambiguous or uncertain situations. For example, in this study, potential influences of gender and age on the differences between LC-MS/MS and CLIA detection methods can be better addressed employing fuzzy logic. This advantage has already been showed in previous research^33^. However, another advantage of fuzzy logic is its ability to adapt inference based on changes in variables without altering the overall model structure^34^. This will be discussed in detail in the next section on the study’s limitations.

The study is limited by the challenges of the sample collection, resulting in certain limitations. The gender imbalance in the samples could introduce slight bias into the results and have a limited impact on the validation of the fuzzy logic analysis. Furthermore, the large number of female samples and their overlap increase the model’s sensitivity to this group, potentially causing overfitting^33^. This phenomenon may be attributed to women’s greater attention to health indicators. According to the China Health and Nutrition Survey^35^, the proportion of women participating in physical examination is significantly higher than that of men, which may be a key factor contributing to the gender imbalance in the samples. The validation results provide preliminary support for the fuzzy logic inference, the aged 30–40 women’s testing differences 30–40 are more pronounced, which may be associated with greater fluctuations in hormone levels during this age range, thereby affecting vitamin D metabolism. Zhao et al.^36^ investigated the impact of sex hormones on vitamin D levels, suggesting that fluctuations in sex hormone-binding globulin (SHBG) levels in women may interfere with the accuracy of vitamin D level measurements. It means that the GENFIS-identified 30–40 female pattern should be interpreted cautiously, as the dataset lacked other affection factors such as BMI, hormonal status, and dietary intake, and the validation set was limited. In future studies, a larger sample size and additional variables should be included to further validate these findings. This study employs fuzzy logic to analyze the variables that are difficult to be handled by traditional methods, such as the potential influence of gender and age on testing differences. These variables lack clear boundaries, and fuzzy logic proves more effective in modeling underlying trends. According to the results of the fuzzy reasoning verification, despite an RR of 3.18 with a 95% CI of 1.71–5.93—meaning the interval excludes 1 (*p* < 0.05) and thus demonstrates statistical significance—further enlarging the sample size would help to strengthen the robustness of the fuzzy logic model’s validation. Future studies should expand collaboration efforts and collect samples through multicenter and perspectives to better train the model, further enhancing the precision and reliability of the results. It will promote advancements in detection technology and contribute positively to the development of precision medicine and public health^37–40^.

This study introduced a novel use of fuzzy logic, to analyze and compare the differences between LC-MS/MS and CLIA in 25(OH)D testing, providing new findings and insights for differential analysis. The data analysis in this study is based on the detection results from the Roche e 801 and AB Sciex QTRAP 6500 instruments. Compared to previous similar studies, these two devices exhibit greater technological advancement. For instance, as mentioned earlier, the AB Sciex QTRAP 6500 is currently the most advanced instrument certified by the CFDA. To the best of our knowledge, these two instruments are at the forefront of medical laboratory platforms in China. This indicates that the results obtained in this study are more precise and accurate, significantly enhancing the reliability of the analytical conclusions and providing a solid foundation for elucidating the differences between the detection methods.

## Conclusion

By broadening the study population and integrating fuzzy logic–based analytical techniques, this research provides a novel perspective on the methodological differences between CLIA and LC-MS/MS for measuring serum 25(OH)D. The results indicate that LC-MS/MS consistently yielded significantly higher values than CLIA (*p* < 0.01), while both methods demonstrated strong linear correlation and high consistency. GENFIS further revealed a distinct pattern in which women aged 30–40 years exhibited greater inter-method discrepancies, suggesting potential demographic influences on measurement variation. By incorporating fuzzy logic into the comparative analysis of two advanced detection techniques, this study enhances both the reliability and interpretability of differential assessment. Nonetheless, the gender imbalance in the sample population introduces a potential source of bias that may influence the conclusions. Future research should therefore focus on achieving more balanced sampling and validating the observed patterns across larger and more diverse cohorts to strengthen the generalizability and robustness of vitamin D detection standardization.

## Supplementary Information

Below is the link to the electronic supplementary material.

Supplementary Material 1

Supplementary Material 2

## Data Availability

All data generated or analysed during this study are included in this published article and its supplementary information files.
