# Supplementary material for: Analysis of chemiluminescence and liquid chromatography-mass spectrometry in 25-hydroxyvitamin D detection using fuzzy logic
Source: Sci Rep. 2026 Mar 3;16:11886. doi: 10.1038/s41598-026-41793-9 (PMC13066485; doi:10.1038/s41598-026-41793-9)
Supplement: Supplementary file 1 — Supplementary Material 1 [file 41598_2026_41793_MOESM1_ESM.docx]

**Appendix：The result of the testing by the two methods**

| **Sample Number** | **CLIA (ng/ml)** | **LC-MS (ng/ml)** | **Difference (ng/ml)** |
| --- | --- | --- | --- |
| **1** | 48.9 | 55.16 | 6.26 |
| **2** | 10.8 | 14.53 | 3.73 |
| **3** | 27.6 | 26.5 | -1.1 |
| **4** | 12.2 | 12.08 | -0.12 |
| **5** | 13 | 15.28 | 2.28 |
| **6** | 19.1 | 18.28 | -0.82 |
| **7** | 27.6 | 27.64 | 0.04 |
| **8** | 16.9 | 18.09 | 1.19 |
| **9** | 18.7 | 24.8 | 6.1 |
| **10** | 33.3 | 43.95 | 10.65 |
| **11** | 16.4 | 23.43 | 7.03 |
| **12** | 9.5 | 8.65 | -0.85 |
| **13** | 8.8 | 8.66 | -0.14 |
| **14** | 24.7 | 31.48 | 6.78 |
| **15** | 27.7 | 40.89 | 13.19 |
| **16** | 22 | 28.96 | 6.96 |
| **17** | 18.4 | 24.69 | 6.29 |
| **18** | 15.7 | 19.73 | 4.03 |
| **19** | 3 | 7.66 | 4.66 |
| **20** | 38 | 44.41 | 6.41 |
| **21** | 27.1 | 31.3 | 4.2 |
| **22** | 12.1 | 9.4 | -2.7 |
| **23** | 14 | 14.07 | 0.07 |
| **24** | 20 | 19.98 | -0.02 |
| **25** | 21.7 | 20.8 | -0.9 |
| **26** | 28.3 | 38.93 | 10.63 |
| **27** | 12.7 | 13.11 | 0.41 |
| **28** | 17.6 | 18.92 | 1.32 |
| **29** | 24.1 | 31.34 | 7.24 |
| **30** | 15.1 | 14.48 | -0.62 |
| **31** | 9 | 8.13 | -0.87 |
| **32** | 27 | 25.23 | -1.77 |
| **33** | 14.9 | 16.89 | 1.99 |
| **34** | 11.9 | 11.43 | -0.47 |
| **35** | 16 | 14.07 | -1.93 |
| **36** | 16.4 | 16.77 | 0.37 |
| **37** | 8.8 | 8.53 | -0.27 |
| **38** | 28.1 | 34.35 | 6.25 |
| **39** | 14.9 | 14.03 | -0.87 |
| **40** | 37.1 | 44.52 | 7.42 |
| **41** | 10.2 | 8.78 | -1.42 |
| **42** | 12.5 | 10.35 | -2.15 |
| **43** | 11.3 | 9.07 | -2.23 |
| **44** | 15.3 | 12.93 | -2.37 |
| **45** | 14.7 | 13.47 | -1.23 |
| **46** | 22.2 | 23.02 | 0.82 |
| **47** | 28.6 | 28.17 | -0.43 |
| **48** | 39.2 | 40.07 | 0.87 |
| **49** | 14.4 | 10.65 | -3.75 |
| **50** | 18.2 | 20.14 | 1.94 |
| **51** | 16.7 | 18.42 | 1.72 |
| **52** | 31.4 | 31.32 | -0.08 |
| **53** | 25.2 | 25.43 | 0.23 |
| **54** | 22.1 | 22.71 | 0.61 |
| **55** | 12.5 | 13.89 | 1.39 |
| **56** | 12.4 | 10.41 | -1.99 |
| **57** | 26.8 | 25.53 | -1.27 |
| **58** | 25.5 | 29.33 | 3.83 |
| **59** | 21.4 | 23.33 | 1.93 |
| **60** | 12.4 | 11.41 | -0.99 |
| **61** | 13.2 | 10.73 | -2.47 |
| **62** | 12.1 | 10.73 | -1.37 |
| **63** | 14.2 | 14.93 | 0.73 |
| **64** | 48.9 | 50.34 | 1.44 |
| **65** | 31.4 | 42.99 | 11.59 |
| **66** | 10.8 | 12.25 | 1.45 |
| **67** | 30.1 | 31.4 | 1.3 |
| **68** | 10.2 | 16.59 | 6.39 |
| **69** | 22.7 | 21.57 | -1.13 |
| **70** | 31 | 41.91 | 10.91 |
| **71** | 16.9 | 16.48 | -0.42 |
| **72** | 30.3 | 38.29 | 7.99 |
| **73** | 7.1 | 5.09 | -2.01 |
| **74** | 28 | 36.22 | 8.22 |
| **75** | 37.6 | 36.14 | -1.46 |
| **76** | 12.2 | 14.63 | 2.43 |
| **77** | 12.8 | 13.12 | 0.32 |
| **78** | 13 | 14.67 | 1.67 |
| **79** | 23.2 | 29.68 | 6.48 |
| **80** | 24.5 | 31.62 | 7.12 |
| **81** | 18.9 | 21.02 | 2.12 |
| **82** | 17.3 | 16.22 | -1.08 |
| **83** | 15.4 | 14.2 | -1.2 |
| **84** | 12.1 | 13.26 | 1.16 |
| **85** | 37.1 | 37.63 | 0.53 |
| **86** | 26.4 | 21.84 | -4.56 |
| **87** | 16.5 | 14.63 | -1.87 |
| **88** | 11 | 7.77 | -3.23 |
| **89** | 30.2 | 27.87 | -2.33 |
| **90** | 23 | 26.9 | 3.9 |
| **91** | 29.8 | 32.27 | 2.47 |
| **92** | 23.8 | 22.53 | -1.27 |
| **93** | 20.5 | 17.55 | -2.95 |
| **94** | 41.8 | 54.44 | 12.64 |
| **95** | 10.3 | 6.77 | -3.53 |
| **96** | 21.5 | 18.7 | -2.8 |
| **97** | 14.6 | 15.52 | 0.92 |
| **98** | 29.6 | 27.91 | -1.69 |
| **99** | 15.2 | 14.92 | -0.28 |
| **100** | 17.4 | 15.24 | -2.16 |
| **101** | 21.1 | 19.4 | -1.7 |
| **102** | 14.8 | 14.61 | -0.19 |
| **103** | 23.8 | 22.6 | -1.2 |
| **104** | 11.2 | 8.2 | -3 |
| **105** | 23.3 | 24.97 | 1.67 |
| **106** | 55.2 | 56.24 | 1.04 |
| **107** | 21 | 19.82 | -1.18 |
| **108** | 13.5 | 10.79 | -2.71 |
| **109** | 26.4 | 23.48 | -2.92 |
| **110** | 30.1 | 33.8 | 3.7 |
| **111** | 25.5 | 29.98 | 4.48 |
| **112** | 13.3 | 10.73 | -2.57 |
| **113** | 13.7 | 13.92 | 0.22 |
| **114** | 29.5 | 31.1 | 1.6 |
| **115** | 11.5 | 11.79 | 0.29 |
| **116** | 24.1 | 23.54 | -0.56 |
| **117** | 11 | 8.84 | -2.16 |
| **118** | 18.5 | 19.58 | 1.08 |
| **119** | 18.4 | 22.12 | 3.72 |
| **120** | 33 | 34.21 | 1.21 |
| **121** | 16.7 | 15.01 | -1.69 |
| **122** | 30 | 26.04 | -3.96 |
| **123** | 10.7 | 10.94 | 0.24 |
| **124** | 31.9 | 36.17 | 4.27 |
| **125** | 32.7 | 38.61 | 5.91 |
| **126** | 22.6 | 28.5 | 5.9 |
| **127** | 11.3 | 10.21 | -1.09 |
| **128** | 21.7 | 24.5 | 2.8 |
| **129** | 14.8 | 13.84 | -0.96 |
| **130** | 23.8 | 23.67 | -0.13 |
| **131** | 15.5 | 12.43 | -3.07 |
| **132** | 8.1 | 7.14 | -0.96 |
| **133** | 21.1 | 23.01 | 1.91 |
| **134** | 11.8 | 12.33 | 0.53 |
| **135** | 32.9 | 39.7 | 6.8 |
| **136** | 31.6 | 31.95 | 0.35 |
| **137** | 17.9 | 18.04 | 0.14 |
| **138** | 19.6 | 24.21 | 4.61 |
